# Diagnostic Challenges of Thyrotoxicosis in Pregnancy Presenting as Stroke and Hyperemesis Gravidarum

**DOI:** 10.7759/cureus.93831

**Published:** 2025-10-04

**Authors:** Varsha Pudi, Kanishka Kamath, Fatima Kamal, Jahangir Rouzbehani

**Affiliations:** 1 Internal Medicine, University of Vermont, Larner College of Medicine, Burlington, USA; 2 Internal Medicine, Danbury Hospital, Danbury, USA

**Keywords:** clinical endocrinology, gestational thyrotoxicosis, graves disease, medical disorders in pregnancy, tachycardia in pregnancy

## Abstract

Hyperthyroidism in pregnancy carries serious risks for both the pregnant individual and the fetus. Presenting symptoms may include fatigue, nausea, and palpitations. Timely diagnosis and intervention are challenging because clinical features overlap with physiological changes in pregnancy. This case report describes a diagnostically challenging presentation of thyrotoxicosis in pregnancy with neurological symptoms mimicking a stroke, which is atypical since stroke-like presentations are not typically associated with hyperthyroidism. This case highlights the importance of maintaining a broad differential in pregnant individuals with persistent, unexplained systemic symptoms.

## Introduction

Hyperthyroidism is an uncommon condition in pregnancy, and the risk of developing Graves’ disease during pregnancy is approximately 0.05% [1]. The overall prevalence of Graves’ disease in the population is 1.2%, most commonly affecting individuals aged 20-50 years [2]. Graves’ disease is the most common cause of hyperthyroidism in pregnancy [3]. The pathophysiology of Graves’ disease involves immunoglobulins that stimulate thyroid-stimulating hormone receptors, leading the thyroid gland to secrete excessive amounts of thyroid hormone. The level of antibodies tends to correlate with disease activity [4].

Current guidelines from the Endocrine Society recommend screening only for pregnant individuals who are at high risk of thyroid disease [5]. This means targeted thyroid screening for pregnant individuals with known risk factors, such as a personal or family history of thyroid disease or clinical signs of dysfunction. However, a study found that targeted testing of only high-risk pregnant individuals would miss about one-third of pregnant individuals with overt or subclinical hypothyroidism [6]. This limitation likely extends to hyperthyroidism as well, highlighting the importance of including thyroid dysfunction in the differential for patients with unexplained systemic symptoms.

Graves’ disease is typically associated with classic symptoms such as sleep disturbances, palpitations, and heat intolerance. However, these symptoms tend to overlap with physiologic changes of pregnancy, making the diagnostic process more complicated. If undiagnosed or untreated, thyrotoxicosis can result in thyroid storm, a life-threatening condition that may involve hyperpyrexia, tachycardia, cardiac arrhythmias, congestive heart failure, vomiting, and neuropsychiatric issues such as restlessness or delirium [7]. This can result in severe complications for the pregnant individual and fetus. Clinically, hyperthyroidism due to Graves’ disease remains an important cause of maternal and fetal morbidity [2]. In fetuses, the most common manifestations are fetal loss, fetal growth restriction, preterm birth, and low birth weight [8]. The fetal thyroid gland begins to function after the first trimester; therefore, extreme fluctuations in maternal T4 levels, particularly during the first trimester, can critically affect fetal neurological development [9].

This case report describes a pregnant woman whose initial presentation of Graves’ disease involved a wide range of cardiopulmonary and neurologic symptoms, highlighting the diagnostic challenge of recognizing thyrotoxicosis in pregnancy.

## Case presentation

A 26-year-old primigravida at 15 weeks of a singleton spontaneous gestation presented to the emergency department following two syncopal episodes at home. She had no significant past medical history. The patient reported persistent nausea and vomiting since the beginning of her pregnancy, which had progressively worsened over the past two weeks.

At presentation, the patient reported intermittent confusion starting that morning, along with constant, sharp, non-radiating chest pain localized beneath the left breast for the past three days. She also endorsed palpitations and shortness of breath. She denied fever and cough. The patient reported a 30-pound weight loss over the course of her pregnancy. On physical examination, the patient was noted to have right-sided horizontal nystagmus, as documented by the emergency department physician; no formal ophthalmology assessment was performed during hospitalization. Her vital signs were remarkable for a blood pressure of 133/57 mmHg and tachycardia at 117 bpm.

In the ED, IV fluids were given to address the patient’s syncope, suspected to be due to dehydration secondary to hyperemesis gravidarum. In addition, the patient was given IV ondansetron and metoclopramide for her hyperemesis. Despite volume resuscitation, the symptoms persisted.

The electrocardiogram showed sinus tachycardia with a ventricular rate of 111 bpm and QTc of 378 ms. No ischemic changes were present, and cardiac troponins were within normal limits.

At presentation, the differential diagnosis was broad due to the persistence of chest pain, dizziness, and shortness of breath, along with right-sided horizontal nystagmus documented by the emergency department physician, despite fluid resuscitation. Given the symptoms and the potential hypercoagulability associated with pregnancy, a pulmonary embolism became a prominent part of the differential. A chest CT angiography with automated exposure control to minimize fetal radiation exposure was performed. Once a large pulmonary embolism was excluded, the patient was admitted to the internal medicine service with working diagnoses of syncope and hyperemesis gravidarum.

**Figure 1:**
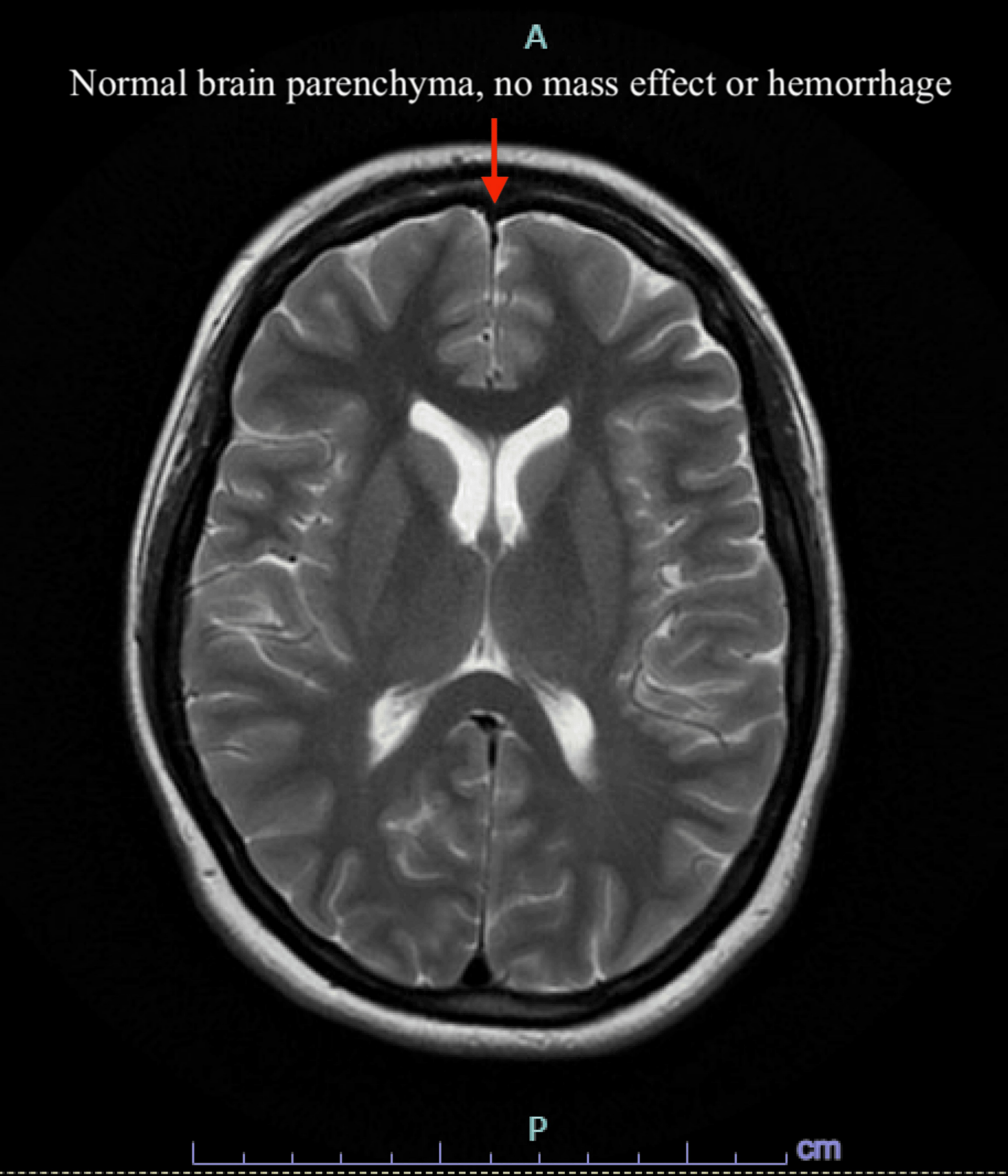
MRI of the Brain Demonstrating No Acute Abnormalities

The patient’s hospital course was complicated by a sudden episode of slurred speech and drowsiness. Upon awakening, the patient was slow to respond and complained of chest pain, left-sided weakness, and decreased temperature sensation in the left extremities. The patient’s stroke-like episode involving slurred speech and unilateral sensory deficits expanded the differential to include a cerebrovascular event. An acute ischemic stroke seemed unlikely due to inconsistent sensory deficits. Magnetic resonance imaging (MRI) without contrast was performed to rule out a structural abnormality, which was unremarkable for infarction, hemorrhage, and structural abnormalities (Figure 1). Although the chest pain exhibited musculoskeletal characteristics from admission, a cardiac etiology was initially considered and ruled out through appropriate evaluation. The consistent features of the pain throughout the course of hospitalization, along with a negative cardiac workup, supported a diagnosis of chest wall or costochondral discomfort related to intractable vomiting.

**Figure 2:**
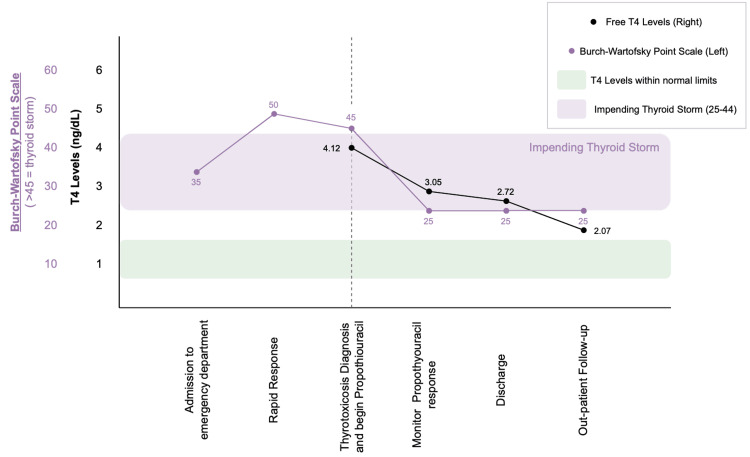
Trend of Free T4 Levels and Burch-Wartofsky Point Scale Through Hospital Course and Treatment The patient’s free T4 levels (black line, right axis) and Burch-Wartofsky Point Scale (purple line, left axis) are shown across key points during the patient’s hospitalization and early outpatient follow-up. The green shaded area represents the reference range for free T4. The purple shaded area represents the reference range for impending thyroid storm per the Burch-Wartofsky Point Scale. The vertical dotted line depicts when a diagnosis of thyrotoxicosis was made and when Propylthiouracil treatment began. Free T4 levels and Burch-Wartofsky scores decreased steadily with treatment, reflecting clinical improvement.

**Figure 3:**
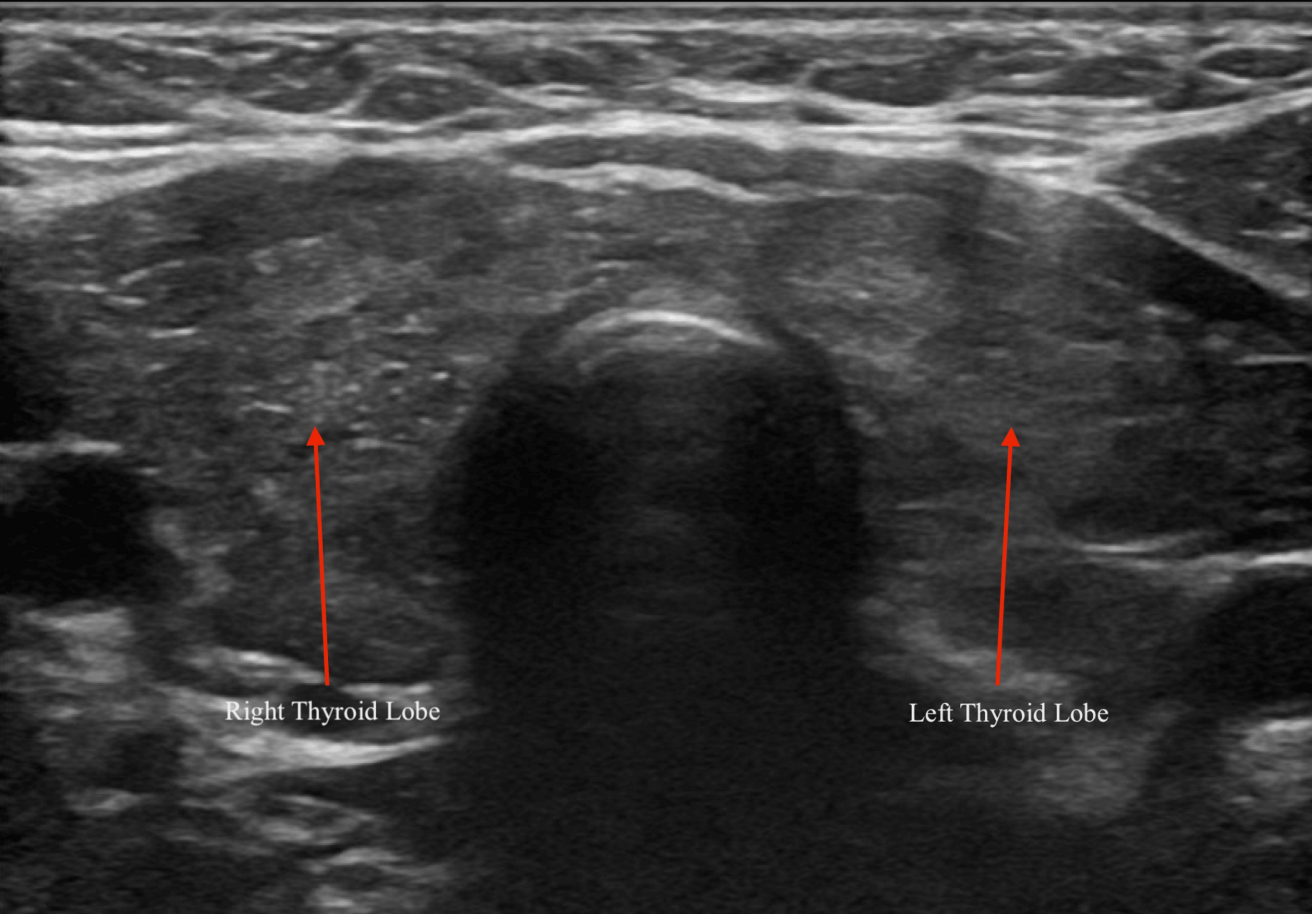
Transverse Thyroid Ultrasound Demonstrating No Abnormalities

In light of the patient’s persistent symptoms, unremarkable imaging, and lack of definitive cardiopulmonary or neurologic findings, a metabolic workup was obtained, including thyroid function tests. This testing revealed a suppressed thyroid-stimulating hormone (TSH) level of <0.01 µIU/mL (reference range: 0.27-4.2 µIU/mL) and a markedly elevated free T4 level of 4.12 ng/dL (reference range: 0.80-1.70 ng/dL), confirming a diagnosis of thyrotoxicosis (Figure 2). A head and neck ultrasound was performed, which showed a heterogeneous thyroid without discrete nodules (Figure 3). Thyroid-stimulating immunoglobulin was 11.50 IU/L, and TSH receptor antibodies were 9.26 IU/L, consistent with Graves’ disease.

The patient continued to report dizziness throughout her hospitalization, including at discharge. Although the symptom persisted, it improved in conjunction with thyroid function, suggesting it was likely attributable to her thyroid dysfunction.

Based on the patient’s thyroid laboratory results, a diagnosis of thyrotoxicosis was confirmed. Once the diagnosis was confirmed, the patient was started on propylthiouracil (PTU) at a dose of 50 mg three times a day. After PTU was initiated, the patient’s free T4 levels began to downtrend. One day after starting treatment, the patient’s free T4 level was 3.05 ng/dL and free T3 level was 7.7 pg/mL. On the day of discharge, the patient’s free T4 level was 2.72 ng/dL and free T3 level was 7.3 pg/mL (Figure 2). She was discharged on PTU with advice to follow up with endocrinology.

The patient remained clinically stable following discharge, with improving thyroid hormone levels and resolution of neurologic symptoms (Table 1). Despite the maternal thyrotoxicosis, the fetus remained stable throughout hospitalization with no clinical manifestations of hyperthyroidism. The patient has been following up with endocrinology on an outpatient basis and has continued to take propylthiouracil. Although a direct causal relationship cannot be established, the resolution of tachycardia and systemic symptoms following medication management for Graves’ disease supports thyrotoxicosis as a likely contributing factor.

**Table 1:** Evolution of Burch-Wartofsky Point Scale and Clinical Features in Suspected Thyroid Storm

Timeline	Average Temperature	Central Nervous System Effects	Gastrointestinal-Hepatic Dysfunction	Average Heart Rate	Congestive Heart Failure	Atrial Fibrillation	Precipitating Event	Burch-Warsofsky Point Scale
Day 1: Admission to Emergency Department	36.7 Celsius +0	Absent +0	Moderate +10	124.6 +15	Absent +0	Absent +0	Present, hyperemesis gravidarum +10	35 Suggestive of impending thyroid storm
Day 2: Rapid Response	36.8 Celsius +0	Moderate +20	Moderate +10	110.2 +10	Absent +0	Absent +0	Present, hyperemesis gravidarum +10	50 Highly suggestive of thyroid storm
Day 3: Thyrotoxicosis Diagnosis and Medication Management	36.8 Celsius +0	Absent +20	Moderate +10	105.6 +5	Absent +0	Absent +0	Present, hyperemesis gravidarum +10	45 Highly suggestive of thyroid storm
Day 4: Medication Management	36.9 Celsius +0	Absent +0	Moderate +10	105.8 +5	Absent +0	Absent +0	Present, hyperemesis gravidarum +10	25 Suggestive of impending thyroid storm
Day 5: Discharge	36.8 Celsius +0	Absent +0	Moderate +10	96.0 +5	Absent +0	Absent +0	Present, hyperemesis gravidarum +10	25 Suggestive of impending thyroid storm

## Discussion

Diagnosing thyrotoxicosis in pregnancy is inherently difficult, especially when symptoms overlap with physiologic pregnancy-related changes. In this case, the constellation of nonspecific symptoms and absence of classical physical exam findings made the diagnosis particularly challenging. The Burch-Wartofsky Point Scale, proposed in 1993, is a tool used to predict the likelihood of impending thyroid storm [10]. It incorporates elements, including precipitating factors and severity of multi-organ decompensation, such as body temperature, central nervous system effects, gastrointestinal-hepatic dysfunction, heart rate, congestive heart failure, and the presence or absence of atrial fibrillation.

One of the most critical features of this case was the extent of neurologic symptoms, including syncope, pseudostroke, and unilateral sensory defects. Thyrotoxicosis has been reported to cause pseudostroke symptoms through thyrotoxic hypokalemic periodic paralysis [11]. In this case, the patient’s potassium levels remained stable throughout admission, making this mechanism unlikely. Thyrotoxicosis is associated with neurologic symptoms such as delirium and extreme lethargy, but the unique unilateral sensory deficits in this case prompted an extensive stroke workup. The inconsistencies of the focal sensory deficits alongside the unremarkable imaging made an endocrine or metabolic etiology more likely.

In this case, elevated thyroid-stimulating immunoglobulin and the presence of thyroid-stimulating hormone receptor antibodies confirmed the diagnosis of Graves’ disease. While Graves’ disease is well studied, its neurologic manifestations are relatively undocumented, particularly during pregnancy. Although the mechanism of the patient’s stroke-like symptoms remains unknown, this could potentially represent a phenomenon known as encephalopathy associated with autoimmune thyroid disease (EAATD). Only fourteen patients with Graves’ disease have been reported to present with symptoms of EAATD [12], and none were pregnant. EAATD can present with stroke-like focal signs, seizures, loss of consciousness, hallucinations, or other symptoms. While corticosteroid treatment is recommended, spontaneous remissions have also been reported [13]. Although a definitive diagnosis of EAATD cannot be made in this case, the transient, unexplained neurologic deficits raise the possibility of EAATD, contributing to the uniqueness and complexity of this patient’s presentation.

When applied retrospectively, the Burch-Wartofsky Point Scale provides additional perspective on the severity of the patient’s hyperthyroidism (Table 1). The patient had moderate abnormalities across several categories, including persistent tachycardia, gastrointestinal symptoms, and neurological changes. The patient’s maximum score on the scale was 50, which is suggestive of thyroid storm (Table 1 and Figure 2). Thyroid storm is a life-threatening condition, and in pregnancy, it can be particularly devastating. The exact mechanisms underlying the progression from uncomplicated hyperthyroidism to thyroid storm remain unclear; however, timely diagnosis and treatment are critical [14].

Initial management of hyperthyroidism in pregnancy requires careful balancing of benefits and risks. Although both propylthiouracil (PTU) and methimazole can elevate liver enzymes, the benefits of controlling maternal thyrotoxicosis outweigh these risks. PTU is generally preferred during thyroid storm in pregnancy because of its added benefit of reducing peripheral conversion of T4 to T3 [6].

## Conclusions

This case highlights the importance of considering endocrine etiologies, specifically thyrotoxicosis, in pregnancy. While some symptoms may mimic typical pregnancy-related changes, a broader differential should be pursued if systemic symptoms are persistent or atypical. A delay in diagnosis could be fatal for both the pregnant individual and the fetus. In this case, the fetus remained stable throughout hospitalization with no clinical evidence of hyperthyroidism, likely due to timely diagnosis and treatment. Additionally, this case demonstrates that careful follow-up and timely medication management can result in clinical stability and symptom resolution. By presenting this case, we aim to underscore the diagnostic challenge of recognizing atypical presentations of Graves’ disease and to encourage clinicians to consider thyrotoxicosis in pregnant individuals with persistent, unexplained systemic symptoms.

## References

[1] Cooper DS, Laurberg P (2013). Hyperthyroidism in pregnancy. Lancet Diabetes Endocrinol.

[2] (2025). Graves disease. https://www.ncbi.nlm.nih.gov/books/NBK448195/.

[3] Mestman JH (1998). Hyperthyroidism in pregnancy. Endocrinol Metab Clin North Am.

[4] Burch HB, Cooper DS (2015). Management of Graves disease: a review. JAMA.

[5] (2025). Hypothyroidism in pregnancy. American Thyroid Association. https://www.thyroid.org/hypothyroidism-in-pregnancy/.

[6] Vaidya B, Anthony S, Bilous M, Shields B, Drury J, Hutchison S, Bilous R (2007). Detection of thyroid dysfunction in early pregnancy: universal screening or targeted high-risk case finding?. J Clin Endocrinol Metab.

[7] Vadini V, Vasistha P, Shalit A, Maraka S (2024). Thyroid storm in pregnancy: a review. Thyroid Res.

[8] Moleti M, Di Mauro M, Sturniolo G, Russo M, Vermiglio F (2019). Hyperthyroidism in the pregnant woman: maternal and fetal aspects. J Clin Transl Endocrinol.

[9] Delitala AP, Capobianco G, Cherchi PL, Dessole S, Delitala G (2019). Thyroid function and thyroid disorders during pregnancy: a review and care pathway. Arch Gynecol Obstet.

[10] Bacuzzi A, Dionigi G, Guzzetti L, De Martino AI, Severgnini P, Cuffari S (2017). Predictive features associated with thyrotoxic storm and management. Gland Surg.

[11] Lin SH (2005). Thyrotoxic periodic paralysis. Mayo Clin Proc.

[12] Tamagno G, Celik Y, Simó R (2010). Encephalopathy associated with autoimmune thyroid disease in patients with Graves' disease: clinical manifestations, follow-up, and outcomes. BMC Neurol.

[13] Querol Pascual MR, Aguirre Sánchez JJ, Velicia Mata MR, Gahete Jiménez C, Durán Herrera MC, González Dorrego F (2000). Hashimoto's encephalitis: a new case with spontaneous remission (Article in Spanish). Neurologia.

[14] Chiha M, Samarasinghe S, Kabaker AS (2015). Thyroid storm: an updated review. J Intensive Care Med.

